# Reliability of HOME FAST BRAZIL—Self-Reported Version for Community-Dwelling Older Adults

**DOI:** 10.3389/fpubh.2021.713202

**Published:** 2021-12-08

**Authors:** Karina Stella Aoki Ferreira, Tamires Terezinha Gallo da Silva, Jarbas Melo Filho, Natacha Verônica Bazanella, Audrin Said Vojciechowski, Lynette Mackenzie, Anna Raquel Silveira Gomes

**Affiliations:** ^1^Occupational Therapy Department, Federal University of Parana, Curitiba, Brazil; ^2^Masters and PhD Program in Physical Education-PPGEDF, Federal University of Paraná, Curitiba, Brazil; ^3^Faculty Inspirar, Curitiba, Brazil; ^4^Maternity Hospital Marieta Konder Bornhausen, Itajaí, Brazil; ^5^Faculty of Health Sciences, University of Sydney, Lidcombe, NSW, Australia; ^6^Prevention and Rehabilitation in Physiotherapy Department, Masters and PhD Programs in Physical Education-PPGEDF, Federal University of Parana, Curitiba, Brazil

**Keywords:** accidental falls, aged, reproducibility of results, risk assessment, environmental hazards, risk factors

## Abstract

**Objective:** Verify the intra- and inter-rater reliability of the HOME FAST BRAZIL—Self-reported version and correlate household environmental risks with the history of falls by community-dwelling older adults.

**Method:** Cross sectional study with 50 community-dwelling older adults who were screened by the cut-off point of the Mini Mental State Exam and replied to the HOME FAST BRAZIL—Self-reported version using two evaluators, on three occasions. The reliability analysis was determined by the Intra-class Correlation Coefficient (ICC), considering ICC > 0.70 as adequate. To test the correlations, the Spearman test was used.

**Results:** The mean age of the participants was 73.2 ± 5.8 years. The inter- rater reliability of HOME FAST BRAZIL—Self-reported version was ICC 0.83 (IC95%, 0.70–0.90) and the Intra- reliability ICC 0.85 (IC95%, 0.74–0.91). A risk of falls was verified in 88% of the sample and four environmental risks presented significant correlations with the history of falls.

**Conclusions:** The HOME FAST BRAZIL—Self-reported version presented adequate reliability for the evaluation of household environmental risks for community-dwelling older adults. Risks such as inadequate armchairs/ sofas, the absence of anti-slip mats in the shower recess, the presence of pets and inadequate beds require attention in the evaluation of household risks, due to their correlation with the occurrence of falls.

## Introduction

Falls are common events amongst the community-dwelling older adults, and can cause injuries and fractures leading to a decline in functional capacity, compromising independence and causing considerable health costs (1–4). The etiology of falls is multifactorial, resulting from the interaction between intrinsic and extrinsic factors, the risk increasing linearly with the number of factors which older adults are exposed to Moreira et al. (5) and Phelan et al. (6). Intrinsic factors are related to dysfunctions in systems that contribute to postural control (sensory, musculoskeletal, and central nervous system) (3).

The environmental risks present in the households of the older adults are linked to the occurrence of falls (1, 7). International guidelines recommend the evaluation of household risks as part of an efficient multifactorial approach for fall prevention (8, 9). Studies have confirmed the effectiveness of household environmental interventions in the reduction of falls by the community-dwelling older adults, and verified that the use of valid instruments that identify environmental risks and circumstances in a standardized way, is one of the fundamental aspects for the prevention of falls (10, 11).

A study found that among the standardized instruments developed to assess the residential risks of falls in the older adults, the Home Fall and Accidents Screening Tool (HOME FAST) (12) is one of the instruments with high potential for evaluating home hazards associated with falls (13). This instrument was translated and culturally adapted to Brazilian Portuguese (14), however, the need for home visits for its application is a limiting factor (15, 16).

To allow the evaluation of the risk of falls without visiting the homes of the community-dwelling older adults, researchers adapted the HOME FAST and developed the *Home Falls and Accidents Screening Tool (HOME FAST) Self-Report Version*, a tool in the English language which allows for the evaluation, in a self-reported way, of household environmental risk factors (17). This tool was translated and culturally adapted for Brazilian Portuguese with the title HOME FAST BRAZIL—Self-report Version (HOME FAST BRAZIL-SR) (18), but its measurement properties have not yet been verified.

One of the measurement properties required to guarantee the quality of an evaluation tool is reliability, which refers to the capacity to reproduce consistent results under different conditions, by the same evaluator or by different evaluators (19, 20).

Thus, the objective of this study was to verify the intra- and inter- rater reliability of HOME FAST BRAZIL—Self-reported version, in Brazilian community-dwelling older adults. Secondly, we aimed to correlate household environmental risks with the history of falls.

## Methods

### Sample Selection

This is a research using a cross-sectional design, developed based on the *Strengthening the Reporting of Observational studies in Epidemiology*—STROBE. Older adults (over 60 years old) of both genders were included, all residents in the community, with adequate visual acuity and with availability to take part in the steps of the research. Individuals who missed one of the research steps and/or who did not reach the cut-off point of the Mini Mental State Exam (Brazilian version) (21) according to their scholastic level (a score of 13 for illiterate people, 18 for those with 1 to 7 years of schooling and 26 for individuals with 8 or more years of schooling), were excluded from the research.

The participants were recruited using convenience sampling, via projects operating within available by the community, and included at least 50 participants according to the sample number recommended for reliability studies (19, 22).

### Ethical Issue

Data collection was carried out by interviews from April to July, 2018, in Curitiba-PR, Brazil, in locations such as the Outpatients Hospital of the Federal University of Parana (while the participants waited to be called in to the Physiotherapy Sector; in a church in the suburb of *Capão Raso* (while the participants waited for physical training made available for the community); and older adults invited for events at SESC in Á*gua Verde* and in *Rua da Cidadania Pinheirinho*. Preliminarily, the invitation to participate in the research was carried out and, in case of acceptance, the older people presented their consent by signing along with the Term of Free and Clarified Consent. The identification data of the participants were filled in, including the full name, gender, age, telephone and scholastic level.

This study was approved by the Ethics in Research Committee of the *Faculdade Pequeno Principe*, Curitiba (PR), Brazil, according to protocol 1.960.06, and by the Ethics in Research Committee of the Municipal Health Secretariat of Curitiba, Curitiba (PR), Brazil, according to protocol 2.083.84.

### Measurements

Information concerning visual acuity adequacy (self-reported) was also obtained and a cognitive selection process applied by way of the Mini Mental State Test. And body mass; height and body mass index (BMI). In addition, an evaluation of the self-reported history of falls was carried out to investigate correlations with environmental risks. The participants were questioned about the occurrence of falls in the last 12 months prior to the evaluation day as well as the number of falls, the place where they occurred and the cause (9).

The risk of falls was evaluated using Home Falls and Accidents Screening Tool Self-report version (HOME FAST-SR) (17), in its Brazilian Portuguese version, HOME FAST BRAZIL-SR (18). The tool has 20 questions subdivided into 97 items, for which the older people participants have to reply “yes” or “no.” The questions evaluate the presence of household environmental dangers, considering mats, floors, steps, objects on the floor, inadequate furniture, lighting, bathroom safety, cupboards, stairs and pets, as well as risky behavior, corresponding to 25 domains derived from Home Falls and Accidents Screening Tool for Health Professionals (HOME FAST-HP) (17). A method has been developed to transform scores from the HOME FAST-SR. i.e., to convert the 97 items to the 25 domains of HOME FAST-HP score. Both the HOME FAST-SR instrument and the transformation method are available at: http://hdl.handle.net/2123/17635. A final score equal or above to 9 indicates a risk of falls in the household (18).

To test the reliability of HOME FAST BRAZIL-SR, the tool was applied to all participants on three different occasions. The tool was applied by two raters (evaluator 1 and evaluator 2) to analyze inter-rater reliability on the same day, independently, with an interval of 40 min. The intra-rater reliability was determined by applying the tool by the same evaluator (evaluator 1) on two different occasions, with an interval of 7 days between applications (23).

### Statistical Analysis

The data were analyzed by descriptive statistics using the Statistical Package for the Social Sciences (SPSS) software, version 20.0, providing the mean, standard deviation and the absolute and relative frequencies. The normality of the data distribution was analyzed by the Shapiro Wilk Test, considering the data normal if *p* > 0.05.

The statistical analysis was run based on the total number of falls, considering both at home and out of home. The *T*-test for Independent Samples was used to compare the variables presenting a normal distribution and for the categorical data was used the Chi-square or Fisher's exact test, considering *p* ≤ 0.05. For the parametric variables, the correlations were tested by the Pearson's Test, and for non-parametric, the Spearman's Test was used, considering *p* ≤ 0.05.

The reliability analysis was determined by the Intraclass Correlation Coefficient (ICC), considering ICC > 0.70 as adequate (23, 24). The Bland-Altman Dispersion Diagram was used to evaluate the magnitude of the differences between the scores obtained in the intra- and inter-rater reliability applications, with the expectation that the values were parallel around the zero horizontal axis and within the confidence limits (23).

## Results

**Figure 1:**
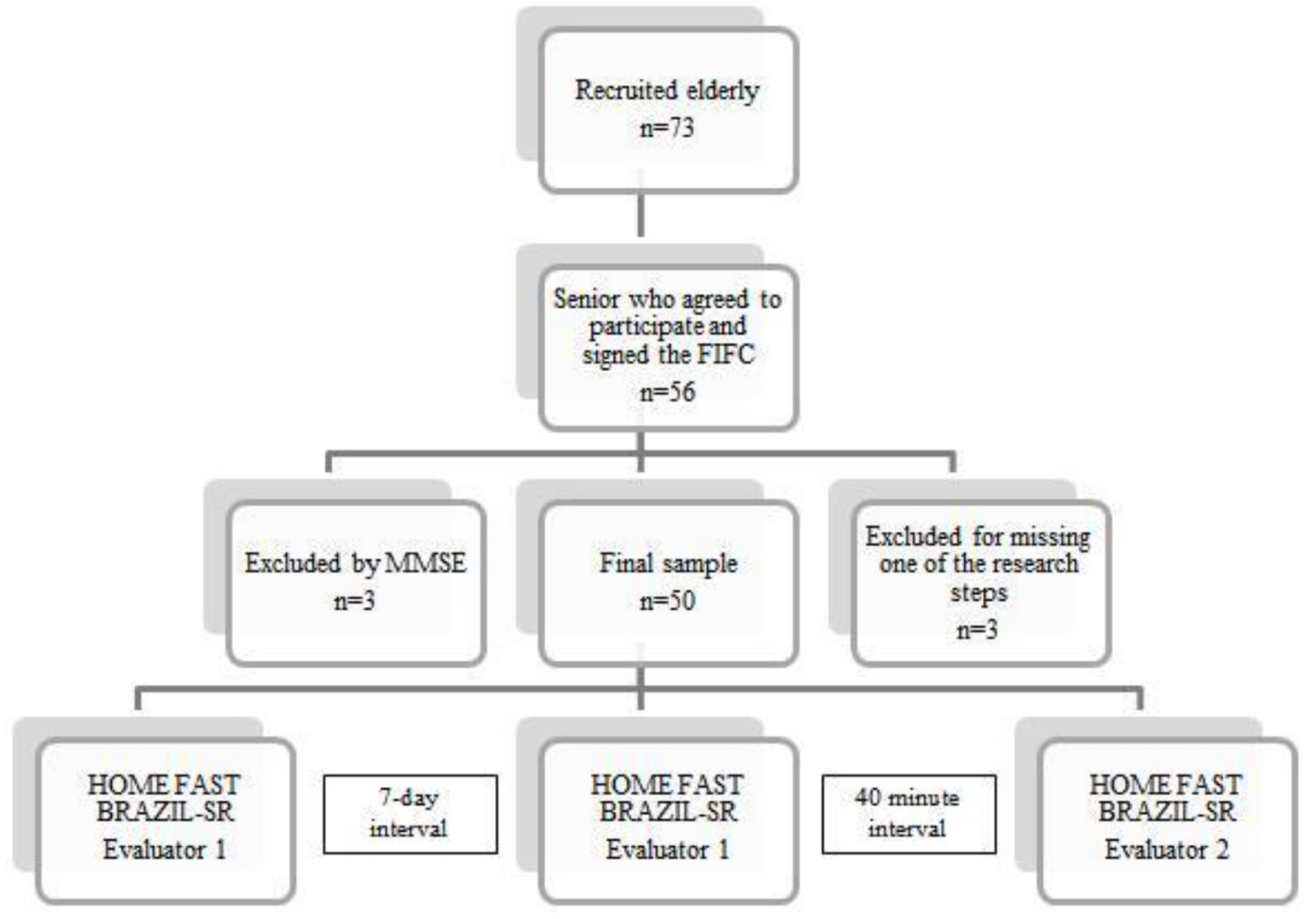
Study flow sheet. *n*, number of people; FICF, Free and Informed Consent Form; MMSE, Mini Mental State Exam.

Figure 1 shows the flow sheet of the study. Seventy-three community-dwelling older adults were invited to participate in the research, of which 17 refused due to a lack of availability. A further 3 were excluded for not reaching the minimum score in the Mini Mental State Exam and a further 3 were excluded because they missed one of the steps of the research. Thus, 50 older adults took part in the study with a mean age of 73.2 ± 5.8 years, of which 84% (*n* = 42) were female and 16% (*n* = 8) male. The sociodemographic characteristics of the participants, divided by gender, can be seen in Table 1.

**Table 1:** Characteristics of the sample.

	**Female (*n* = 42)**	**Male (*n* = 8)**	**Total (*n* = 50)**	***p***
Age (years)^†^	72.92 ± 5.80	75.12 ± 6.05	73.28 ± 5.83	0.33
Stature (m)^†^	1.57 ± 0.06	1.68 ± 0.03	1.59 ± 0.07	0.00^*^
Body mass (kg)^†^	68.47 ± 13.73	79.82 ± 11.02	70.29 ± 13.88	0.03^*^
BMI (Kg/m^2^)^†^	29.12 ± 7.34	30.23 ± 5.98	29.30 ± 7.09	0.68
Education^‡^				
Incomplete elementary school	57.1% (*n =* 24)	75.0% (*n =* 6)	60% (*n =* 30)	0.84
Complete primary education	7.1% (*n =* 3)	–	6% (*n =* 3)	
Incomplete high school	2.4% (*n =* 1)	–	2% (*n =* 1)	
Complete high school	23.8% (*n =* 10)	12.5% (*n =* 1)	22% (*n =* 11)	
Complete high school	9.5% (*n =* 4)	12.5% (*n =* 1)	10% (*n =* 5)	

*BMI, Body Mass Index*;

† *mean values ± standard deviation compared by T-test for Independent Samples*;

‡ *Relative (%) and absolute (number) frequency values by chi-square or Fisher's exact test*.

* *Significant difference*.

**Figure 2:**
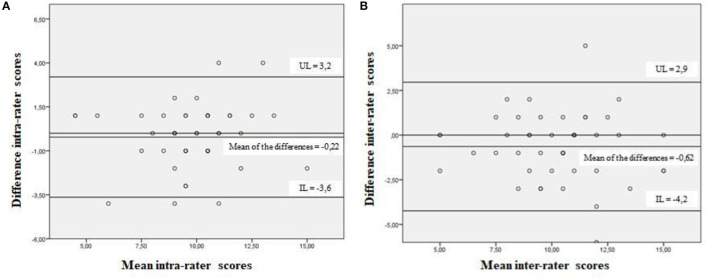
Bland-Altman dispersion diagram with the magnitude of variability between the intra-rater **(A)** and inter-rater **(B)** measurements of the HOME FAST BRAZIL-SR. Curitiba, Paraná, 2018. UL, upper limit; IL, inferior limit.

The scores obtained for the HOME FAST BRAZIL-SR at the three evaluation occasions were: evaluator 1 (9.9 ± 2.4); evaluator 2 (10.6 ± 2.5); evaluator 1 after 7 days (10.2 ± 2.3). The Inter-reliability was ICC 0.83 (0.70–0.90) and the Intra-evaluator reliability was ICC 0.85 (0.74–0.91). Figure 2 presents the Bland Altman Dispersion Diagrams, which show the magnitude of the variability between the intra- and inter-rater reliability measurements using the HOME FAST BRAZIL-SR.

Regarding the history of falls, 18 participants (36%) reported at least one fall in the last year. Of the total number (18; being 17 women fallers and 1 men faller; with average age of 74.61 ± 5.67 years old; stature 1.56 ± 0.06 meters; body mass 70.21 ± 15.68 kg; BMI 28.86 ± 4.96 kg/m^2^; and 66.7%(*n* = 12) with incomplete elementary school) of falls reported, 54.8% occurred on roads and 45.1% in the participant's own home. Self-reported factors responsible for these falls were, 67.7% occurred due to environmental risks (stumbling or slipping); 25.8% due to intrinsic factors (loss of balance or dizziness); and 6.4% due to behavioral factors (climbing up onto a stool or steps to carry out domestic chores).

**Figure 3:**
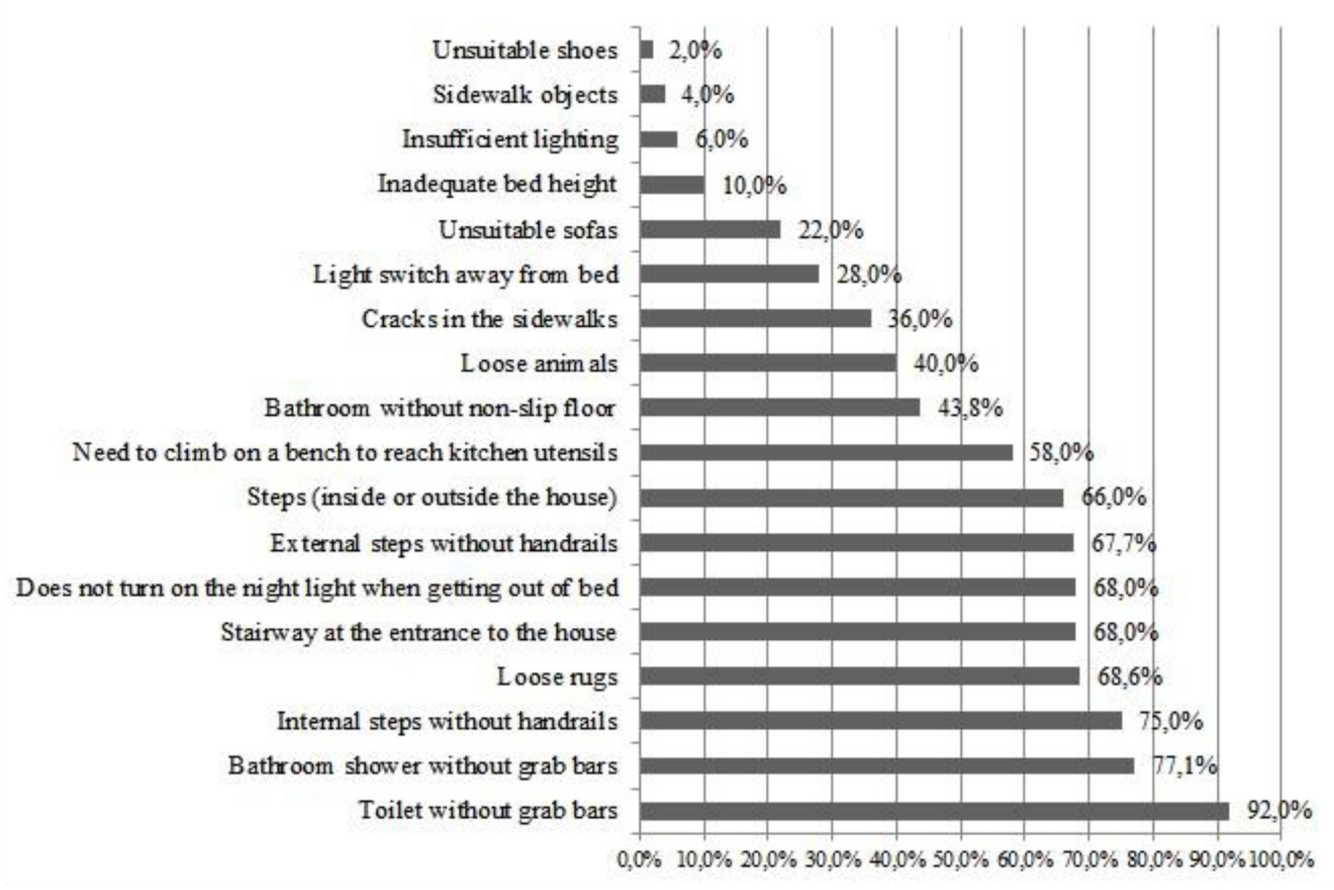
Percentage (%) of risks identified using HOME FAST BRAZIL-SR. Curitiba, Paraná, 2018.

The mean score for the evaluation of the risk of falls according to the HOME FAST BRAZIL-SR was 9.98 ± 2.41, being 9.7 ± 2.4 for females and 11 ± 2.3 for males, with no significant difference between genders (t:11.34; *p* = 0.208, *t-test for independent samples*). A total of 44 of the participants (88%) presented a score equal or >9 indicating a high risk of falls. The frequency of hazards related to falls reported by the participants, through the application of HOME FAST BRAZIL-SR, are shown in Figure 3.

The following environmental risks showed significant correlations with the number of falls that occurred in households: presence of inadequate armchairs/sofas (*r* = 0.76; *p* = 0.01, *Spearman*), absence of an anti-slip mat in the shower recess (*r* = −1.00; *p* < 0.001, *Spearman*) and needing various attempts to manage to get up from the armchair/sofa (*r* = 0.66; *p* = 0.03, *Spearman*). The following risk showed significant correlation with the general number of falls (in the household and on the street): needing more than one attempt to get up from bed (*r* = 0.47; *p* = 0.04, *Spearman*). The history of falls (in the last year) also showed a significant correlation with the presence of pets in the household (*r* = 0.40; *p* = 0.003, *Spearman*).

## Discussion

The reliability of HOME FAST BRAZIL-Self-reported version was tested in Brazilian community-dwelling older adults and the results indicated that the tool had adequate intra and inter rater reliability to evaluate household environmental risks. Studies that evaluate the measurement properties of evaluation tools with respect to health, such as reliability, provide important information concerning the quality of the tool and determine the choice for application by health researchers and professionals (20, 23). For a test to be considered reliable, it should provide precise and reproducible information by both the same examiner—intra-rater reliability and different examiners—inter-rater reliability (21). In the present study, the results obtained in the verification of the intra-rater and inter-rater reliability confirmed the capacity of HOME FAST BRAZIL-SR to be reproducible both by the same evaluator on distinct occasions and by different evaluators. Good to excellent test-retest reliability was demonstrated for HOME FAST BRAZIL-SR, filling an important gap of the literature, considering that it is the only published self-assessment of the home environment's factors for falls in Brazilian Portuguese. Identifying home hazards by self-report has the potential for older people to increase their awareness of the safety of their home environment, which may lead to more immediate negative effects, such as an older person feeling more vulnerable in their home to plan the changes (17).

Considering the recommendations based on evidence for the multidimensional evaluation of the health of older adults (25), the HOME FAST BRAZIL-SR stood out as an important tool to optimize the screening process for the risk of falls and identify Brazilian community-dwelling older adults at risk, making it possible to prevent future health problems. Falls are amongst the main threats to the health of the older people, being responsible for 95% of hip fractures (3), a fact which made the need for early risk screening urgent.

The present study found a high prevalence of risk, since 88% of the older adults evaluated scored 8 or above in the HOME FAST BRAZIL-SR, the mean score found being 9.9. Similar risk levels were found earlier, where a mean score of 9.4 was found using the same tool in a sample of 568 community-dwelling older adults in Australia (17).

The household fall risks found in the present research are aligned with other research findings (7, 17, 18, 26), the majority being difficult to modify. Earlier studies (6, 27) emphasized the need for intervention in modifiable risk factors for falls, showing that the installation of handrails on stairs, grab bars in bathrooms and non-slip ground coverings in external areas reduced the injury and fracture rates caused by falls.

In the present study, risks such as inadequate armchairs/sofas, the absence of anti-slip mats in shower recesses, the presence of pets and inadequate beds stood out for their association with the occurrence of falls. The number of dangers in a household indicates the degree of exposure of a person to the possibility of falling and the risks associated with falls deserve attention, since they could be contributing to the occurrence of falls (18).

An earlier study showed that the presence of pets in the main entrance to a home was significantly associated with the occurrence of falls in the older adults and increased the chance of falling by a factor of two (28). Risks involving pets were reported by 85.6% of the sample of 567 community-dwelling older adults (18). In the present study, 40% of the older adults presented risks related to the presence of pets, a fact correlating with the history of falls (having fallen during the previous year). These data are a sign of the need to pay more attention to this aspect in the evaluation of community-dwelling older adults so that preventative strategies can be developed.

According to the present study, the older adults who reported difficulty in getting up from bed or from armchairs/ sofas, requiring more than one attempt in order to stand up, suffered more falls in their own homes. Not managing to get up from the bed or from armchairs/ sofas on the first attempt is as much related to a possible inadequacy of the furniture as to mobility alterations in the older person (12). Aging is associated with a reduction in skeletal muscle mass and in muscle strength and power, especially that of the knee extensor muscles, in which a 20 to 40% reduction in strength and power up to the seventh decade of life has been found. This interferes in mobility, functional capacity, and the satisfactory carrying out of sitting down and getting up activities (29, 30). Older people who show the worst results in the sit down and get up from a chair in 30 s test, present inferior functional performance in the carrying out of their daily life activities and their instrumental daily life activities (31). Therefore, we suggest that future studies should carry out physical-functional performance evaluations together with the HOME FAST BRAZIL-SR in order to clinically verify if the strength and power of the lower limbs require physical training to increase the capacity to sit down and get up from bed or from armchairs/ sofas.

In the present study, the older adults who did not use an anti-slip mat in the bathroom referred to a larger number of falls inside their homes. Earlier studies reported the absence of anti-slip mats in the households of community-dwelling older adults as being prevalent (18, 28). The existence of slippery floors in the homes of the older adults is a factor of risk that should be identified and eliminated, since it is implicated in falls and fractures (32).

The epidemiological aspects of falls were described in a study with 324,060 community-dwelling older adults in the State of Victoria, Australia, showing that 80.7% of falls were caused by environmental risks and the home was the most common location for this to occur (33). In the present study, environmental risks were involved in the majority (67.7%) of the falls reported by the community-dwelling older adults, and were responsible for half (50%) of the falls occurring in the person's own home and for the great majority (82.3%) of the falls occurring in the road. Similar results were found in an earlier study (34) which showed that the majority of falls in external environments were caused by environmental risks due to tripping and slipping on sidewalks, curbs and irregular surfaces. Generally, falls occurring in the road or in external environments are related to environmental aspects, whereas falls occurring in the home are more related to the individual's state of health (34–36). The older people who fall in their own homes present worse physical performance and functional mobility as compared to those who fall in the road when tests such as the Timed Up and Go test (TUG), Berg's Equilibrium Scale, Short Physical Performance Battery (SPPB) and Gait velocity are used (36, 37). Thus, the need for measures that improve accessibility in the community are apparent, since the majority of falls that occur in the road are caused by modifiable factors, the implementation of actions that eliminate risks involving sidewalks, curbs and roads being fundamental, with the restoration of irregular surfaces, removal of obstacles and installation of ramps (34, 37).

Therefore, indoor and outdoor falls are a complex event and occur when environmental hazards, tasks or demands exceed the individual's ability to maintain postural control. Due to this complexity, any falls prevention interventions need to take a multi-factorial, individually tailored approach to address a range of risk factors (18).

In addition to the environmental and intrinsic factors of risk, the present study found a high frequency of behavioral risks, such as not switching on a light when getting up at night (68%), and getting up onto a stool or other object to reach utensils for use in the kitchen (58%). Apart from these, 14.2% of the falls occurring in the person's own home were caused by attitudes such as getting up onto a stool or portable steps when carrying out domestic chores. Risky behavior includes attitudes adopted by the community-dwelling older adults which expose them to a greater chance of falling (12, 38). The items in the HOME FAST BRAZIL-SR cover risk categories such as environmental factors, functional factors and behavioral factors (17). Evaluations of household falls should include the investigation of behavioral factors, since domestic safety involves both the nature of the physical characteristics of the environment and the way in which a person interacts with it (12).

The present study has strengths that should be emphasized: HOME FAST BRAZIL-SR is the only self-reported instrument for assessing domiciliary risk of falls in the older people translated and adapted transculturally into Brazilian Portuguese that that presents adequate reliability, assuring its ability to reproduce consistent results by the same evaluator or by different evaluators. Moreover, the data showed that environmental hazards such as inadequate armchairs/sofas, the absence of anti-slip mats in the shower recess, the presence of pets and inadequate beds correlated with the occurrence of falls, contributing to health professionals to guide older people to determine changes, prevent, and diminish falls risk inside home, even without a previous visit at the residence.

The limitations of this study refer to the nature of HOME FAST BRAZIL-SR, by which the identification of household risks is carried out by self-reporting, which can suffer the influence of memory. The sample of the present study was mainly female, there were more women than men at the places where the sample was recruited. Thus, it can be suggested for future studies to balance the genders in the sample, and/or consider a proportionality according to recent demographic statistics. The little number of participants that experienced at least a fall in their home might be considered a limitation. Other measurement properties of the tool still need to be established in Brazil, such as the criterion validity and internal consistency (20). In addition, since this study was of a cross-sectional nature, a cause and effect relationship could not be established, and hence it could not be affirmed that the household environmental risks were modified after the occurrence of falls. Hence the authors suggest the carrying out of a longitudinal study using HOME FAST BRAZIL-SR, to produce evidence concerning the relationship between household environmental risks and the incidence of falls.

## Conclusion

The HOME FAST BRAZIL—Self-reported Version presents adequate intra and inter-rater reliability, and can be used in clinical evaluations and in Brazilian research to identify older adults at risk for household falls. A risk of falls was found in 88% of the sample studied due to the presence of hazards in their households. Amongst the risks identified, the high frequency of the absence of support bars at the side of toilets, the absence of support bars in shower recesses, the absence of handrails on internal steps and loose mats stood out. Risks such as inadequate armchairs/ sofas, the absence of anti-slip mats in shower recesses, the presence of pets and inadequate beds also deserve attention in the evaluation of household risks, since they have shown correlation with the occurrence of falls.

## Data Availability Statement

The raw data supporting the conclusions of this article will be made available by the authors, without undue reservation.

## Ethics Statement

The studies involving human participants were reviewed and approved by Research Ethics Committee of the Pequeno Príncipe College, Curitiba, Paraná, Brazil (number 1.960.069/2017), and by the Research Ethics Committee of the Health Department of the City of Curitiba, Curitiba, Paraná, Brazil (number 2.083.841/2017). The patients/participants provided their written informed consent to participate in this study.

## Author Contributions

KF, NB, JM, AV, LM, and AG participated in the conception, design, analysis, and interpretation of data. KF and TG collected the data. AG participated in the conception and study design, drafting the article, and revising it critically. The authors read and approved the final manuscript.

## Funding

This study was financed in part by the Coordenacão de Aperfeicoamento de Pessoal de Nível Superior - Brasil (CAPES) - Finance Code 001; Residence fellowship from the Brazilian Ministry of Health; Productivity Research fellowship from the National Council for Scientific and Technological Development (CNPq) and Pró-Reitoria de Pesquisa e Pós-graduação (PRPPG) at the Federal University of Paraná (UFPR) for the payment of the publication fee.

## Conflict of Interest

The authors declare that the research was conducted in the absence of any commercial or financial relationships that could be construed as a potential conflict of interest.

## Publisher's Note

All claims expressed in this article are solely those of the authors and do not necessarily represent those of their affiliated organizations, or those of the publisher, the editors and the reviewers. Any product that may be evaluated in this article, or claim that may be made by its manufacturer, is not guaranteed or endorsed by the publisher.
